# Safe practices for bed bathing in the intensive care unit: validation of a checklist

**DOI:** 10.1590/0034-7167-2023-0135

**Published:** 2023-12-08

**Authors:** Juliana Cristina Silva, Márcia Marques dos Santos Felix, Rosa Helena Aparecida Gonçalves, Isadora Braga Calegari, Maria Beatriz Guimarães Raponi, Maria Helena Barbosa

**Affiliations:** I Universidade Federal do Triângulo Mineiro. Uberaba, Minas Gerais, Brazil; II Universidade Federal de Uberlândia. Uberlândia, Minas Gerais, Brazil

**Keywords:** Bath, Checklist, Intensive Care Unit, Bedrindden Persons, Patient Safety, Baños, Lista de Verificación, Unidad de Terapia Intensiva, Paciente Encamado, Seguridad del Paciente, Banho, Lista de Verificação, Unidade de Terapia Intensiva, Paciente Acamado, Segurança do Paciente

## Abstract

**Objective:**

To validate a checklist for safe bathing in critically ill patients.

**Methods:**

This is a methodological and quantitative study. Researchers developed a checklist for safe bathing in critically ill patients consisting of 41 items, which were submitted to the apparent and content validation process, evaluated by eleven judges, and interobserver reliability. For reliability analysis, the instrument was applied in 54 bed bath procedures in the ICU; Kappa and CHF tests were used.

**Results:**

In the apparent and content validation, adjustments were made according to the judges’ suggestions. Kappa values ranged from moderate to almost perfect (0.462 to 0.962), and, in some items, there was 100% agreement; the reliability of the instrument was excellent (ICC = 0.962).

**Conclusion:**

The instrument proved to be dependable and easy to apply. Its use will contribute to safe bed bathing and subsidize interventions aimed at increasing the quality of care.

## RESUMO

**Objetivo:**

Validar uma lista de verificação para o banho seguro em pacientes críticos.

**Métodos:**

Estudo metodológico e quantitativo. Elaborou-se uma lista de verificação para banho seguro no paciente crítico composta por 41 itens, que foi submetida ao processo de validação aparente e de conteúdo, avaliada por 11 juízes, e confiabilidade interobservadores. Para a análise de confiabilidade, o instrumento foi aplicado em 54 procedimentos de banho no leito em UTI; utilizaram-se os testes de Kappa e ICC.

**Resultados:**

Na validação aparente e de conteúdo, foram realizados ajustes conforme sugestões dos juízes. Os valores de Kappa variaram de moderado a quase perfeito (0,462 a 0,962); e, em alguns itens, houve 100% de concordância; a confiabilidade do instrumento foi excelente (ICC = 0,962).

**Conclusão:**

O instrumento demonstrou-se confiável e de fácil aplicação. Sua utilização contribuirá para o banho no leito seguro e subsidiará intervenções que visem aumentar a qualidade assistencial.

## RESUMEN

**Objetivo:**

Validar lista de verificación para el baño seguro en pacientes críticos.

**Métodos:**

Estudio metodológico y cuantitativo. Se elaboró una lista de verificación para baño seguro en paciente crítico compuesta por 41 ítems, que fue sometida al proceso de validación aparente y de contenido, evaluada por 11 jueces, y confiabilidad interobservadores. Para el análisis de confiabilidad, el instrumento fue aplicado en 54 procedimientos de baño en el lecho en UTI; utilizados las pruebas de Kappa e ICC.

**Resultados:**

En la validación aparente y de contenido, fueron realizados ayustes conforme sugestiones de los jueces. Los valores de Kappa variaron de moderado a casi perfecto (0,462 a 0,962); y, en algunos ítems, hubo 100% de concordancia; la confiabilidad del instrumento fue excelente (ICC = 0,962).

**Conclusión:**

El instrumento se demostró confiable y de fácil aplicación. Su utilización contribuirá para el baño en el lecho seguro y subsidiará intervenciones que objetiven aumentar la calidad asistencial.

## INTRODUCTION

Scientific and technological advances in health have brought new possibilities for treatment, cure, and prevention, and the increasing complexity of procedures has aroused researchers’ interest, especially in studies focusing on safety and quality of care.

During hospitalization, due to the complexity of hospital services, patients become more exposed to risks and consequently more vulnerable to damage, which can directly impact their recovery. From this perspective, patient safety should be present in all interventions performed during hospitalization, aiming at solving problems for rapid health recovery and reducing the rates of avoidable patient safety incidents^(1)^. Although some procedures incorporated into the nursing care routine, such as bed bathing, are considered simple in stable patients, they can become complex in the context of critical patients^(2-3)^.

According to Resolution nº 2,271/2020, which regulates the criteria for the operation of intensive care units (ICU) and intermediate care units (IMCU) in Brazil, “critical patient” is defined by a patient who presents one or multiple acute failures of vital organs or is at risk of developing them, with an immediate threat to life and need for high complexity support^(4)^. Critically ill patients hospitalized - especially in the ICU, with long stays and use of broad-spectrum antimicrobials - are at considerable risk of skin colonization by pathogens associated with health care, with a higher probability of subsequent infection^(2)^.

The characteristics of the critical patient make them vulnerable to interventions carried out by the nursing team, and attention is needed since, when unplanned and executed without technical and scientific rigor, they can aggravate the patient’s condition^(5)^.

One of these interventions is bed bathing, which, in the hospital context in which it is performed, places the patient as a passive individual subject to the execution of the procedure by the nursing team^(6)^. Such intervention can induce fall and displacement of devices, causing bloodstream infections. In the United States, this type of infection results in approximately 28,000 deaths, generating additional annual costs for the health system in the billions of dollars^(7)^.

Bed bathing is often neglected because it is a routine activity of the nursing team^(8-9)^. However, it is an essential procedure, as it reduces the risk of infection by reducing the microbial load on the skin, provides comfort to the patient, reduces the appearance of lesions, and allows the nurse to perform a complete and efficient physical examination, subsidizing the nursing process^(10-11)^. It is crucial that nurses not only consider the technique applied, but also be able to evaluate and meet the patient’s care demands in a biopsychosocial way^(12)^.

Studies indicate the possibility of hemodynamic changes during and up to one hour after bed bathing in these patients, such as desaturation, intracranial hypertension, heart rate, blood pressure and respiratory rate changes, ventricular fibrillation and cardiac arrest, and other adverse events, such as disconnection of the mechanical ventilator^(3,13-14)^. Thus, in this context, bed bathing can be considered an activity that offers risks and can lead to clinical instability in these patients^(15)^.

In addition to physiological changes and adverse events, a study conducted in Alexandria, Egypt, described as factors affecting bed bathing in critically ill patients: financial resources, adequate equipment, lack of knowledge, and workload^(16)^.

Some studies indicate that the lack of standardization of evidence-based procedures is related to the practice of unsafe acts during nursing care^(17)^. Scholars in the field point to the relevance of this theme and the importance of new investigations on bed bathing in critically ill patients, given the qualification of care^(9)^ and the existing gaps^(15,18)^.

## OBJECTIVE

To design and validate a checklist for safe bathing in critically ill patients.

## METHODS

### Ethical Aspects

The research was conducted in accordance with resolution 466/12 of the National Health Council. The project was approved by the Research Ethics Committees (REC), data collection field and University linked to the study. All participants signed the Informed Consent.

### Design, period, and place of the study

This is a methodological and quantitative study, developed in two stages: Stage I - elaboration and apparent validation and content of the instrument (checklist); and Stage II - pre-test and interobserver reliability analysis.

The instrument was developed based on the recommendations of the American Association of Critical-Care Nurses^(19)^, in section No. 4 of the National Health Surveillance Agency^(20)^ and evidence available in the literature on bed-bathing practices in critically ill patients^(16,21)^.

In this study, this instrument was called “Checklist for safe bathing in critically ill patients” and underwent apparent and content validation, pretesting and interobserver reliability analysis.

For the apparent and content validation, the instrument was presented to the appreciation of 11 judges, selected through the curriculum on the Lattes platform, considering the degree (Doctor), nursing performance with critical patients, and nursing fundamentals. The study considered a level from 80% agreement among the suggestions of the judges for inclusion/modification of items^(22-23)^.

After the suggested adjustments and to evaluate the operational suitability, the instrument underwent a pre-test, performed with the observation of ten bed bath procedures in critically ill patients. Adjustments were then made to the instrument.

The checklist was presented in its last version, consisting of 41 items, divided into three parts: Part I comprised the actions performed before bathing; Part II, the actions during bathing; and Part III, the actions after bathing. All items of the instrument were organized in the template checklist, with the alternatives for marking each item as: “Yes,” “No” and “does not apply,” in which the “Yes” has Weight 1, “No” is worth 0, and “does not apply” is not counted. Thus, the instrument can generate an adhesion score obtained from the following formula:


$$\text{ Total adherence score }=\times \frac{\sum \text{ of posittive responses }(1)}{\text{ (Total nº of Items -nº of items that do not apply)}}100$$

The last version of the instrument was submitted to interobserver reliability analysis performed by two nurses working in the intensive care unit (ICU), one of them a master’s student and researcher of this study, and the other a nurse worked in the Cardiological ICU.

The study used an instrument containing sociodemographic and clinical data to characterize the sample: sex, age, score in the *S*implified Acute Physiology Score III (SAPS III), drugs in continuous infusion (sedatives, analgesia, vasopressors, and vasodilators), invasive and external devices used. This characterization instrument was developed by the authors and validated by three judges with doctoral degrees.

Observations of bed bath procedures were performed in patients admitted to the general ICU in the period from October to December 2020 in the ICU of a large public Teaching hospital in Minas Gerais to obtain the data.

### Data analysis and statistics

The study calculated the equivalence or agreement index for the reliability analysis to assess interobserver consistency. The Kappa coefficient and the ICC determined the values^(24-25)^. The research calculated the proportion of agreement among the observers and used the descriptive statistics to analyze of categorical variables and measures of central tendency and variability for quantitative variables analysis.

### Sample and criteria of inclusion and exclusion

The calculation of the sample size for interobserver reliability analysis considered an expected intraclass correlation coefficient of ICC = 0.80 between the scores. It should not be lower than ICC = 0.60 for a power of 90%, considering a significance level α = 0.05. With these aprioristic values, using the application Power Analysis and Sample Size (PASS), version 13, a minimum sample size (*n*) of 54 bed bath procedures in critically ill patients, which were selected according to the inclusion criteria.

The study included body hygiene procedures that configured bed baths and excluded those involving only the intimate hygiene of patients.

## RESULTS

The stage of apparent validation and content of the instrument was conducted by eleven judges with Ph.D. degree in the area, nine from the State of Minas Gerais, one from Bahia, and one from the State of Rio Grande do Sul.

They evaluated the first version of the instrument and made suggestions regarding the semantics, structure, and arrangement of the items. Most agreed on the suggestions, considering them pertinent and incorporating them into the instrument to generate its second version. The suggestions are described in the Chart 1.

**Chart 1:** Apparent and content validation of the instrument: suggestions from the judges, Uberaba, Minas Gerais, Brazil, 2021

Suggestions from judges
1 - Description of items in detail - changes of verbs/nomenclature
2 - Measurement of cuff pressure and airway aspiration as a nurse’s private
3 - Nurse guidelines for staff
4 - Specification that the soap used is liquid
5 - Division of items into three parts: before bathing, during bathing and after bathing
6 - Performance of the bath by at least two professionals
7 - Pause of the enteral diet
8 - Use of PPE
9 - Conducting the bath in stages and drying the patient

**Figure 1:**
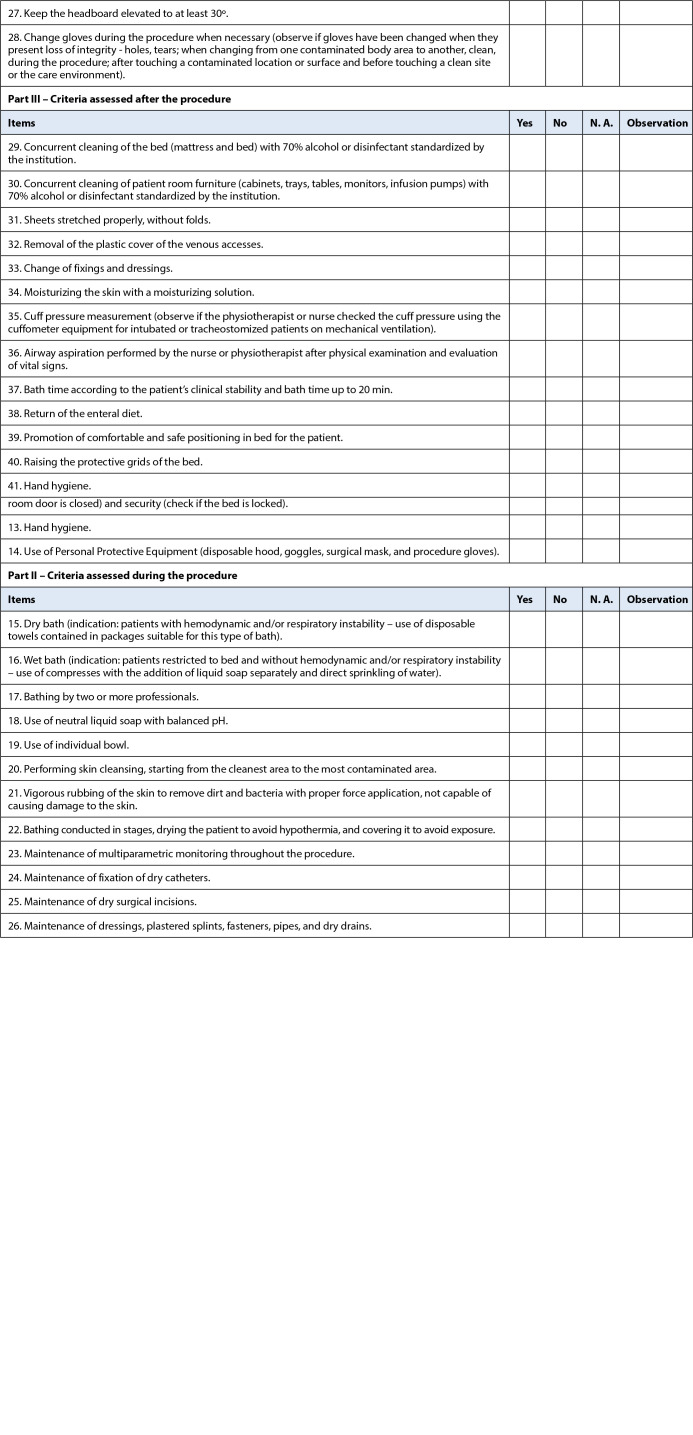
Checklist for safe bathing in critically ill patients (CSBCP). Uberaba, Minas Gerais, Brazil, 2021

This version of the instrument was submitted to the pre-test stage to evaluate the operational suitability; then, the need for adjustments was verified. After being conducted, the last version of the instrument was obtained (Figure 1). There was a readjustment of four actions, only with the dismemberment of the items, without insertion of new ones, thus obtaining items 10 and 11; 13 and 14; 32 and 33; and 38 and 39.

For interobserver reliability, 54 bed bath procedures were observed in critically ill patients using the last version of the instrument. Of this sample, 19 (35%) patients were in the age group of 61 to 70 years, and 29 (53.7%) were male.

As for the risk of mortality, measured by the SAPS III instrument, 15 (27.8%) patients had a score between 61% and 80%; and, in relation to the duration of the procedure, 27 (50%) of the baths lasted from 21 to 40 minutes.

The most used sedatives were midazolam and propofol used by 20 (37%) and 19 (35.2%) patients, respectively. The analgesic of choice was fentanyl (16; 29.6%). The most used vasoactive drug was norepinephrine (23; 42.6%), followed by sodium nitroprusside (6; 11.1%).

The most used invasive devices were central venous catheter (47; 87%), Gastroenterol catheter (45; 83.3%), bladder catheter (43; 79.6%), orotracheal tube (38; 70.4%) and arterial catheter for invasive pressure (29; 53.7%).

The study calculated the proportion of the agreement and the Kappa for each item of the instrument to analyze the agreement between observers. Most of the items presented agreement strength almost perfect, above 81%, a minimum of 72.22%, and maximum of 100%, demonstrating that the items of the instrument were understandable and dependable when applied to the observed context.

Kappa coefficient values ranged from moderate to almost perfect (0.462 to 0.962; p <0.001). Items with 100% agreement, the Kappa coefficient was not calculated since perfect agreement occurred. The proportion of agreement of the items is presented in Table 1.

**Table 1:** Analysis of interobserver reliability of the instrument Checklist for safe bathing in critical patients. Uberaba, Minas Gerais, Brazil, 2021

Items	Observer 1			Observer 2			Proportion of agreement (%)	Kappa	*p* value
	Yes n (%)	No n (%)	The^*^ n (%)	Yes n (%)	No n (%)	The^*^ n (%)			
1	13 (24.0)	41 (76.0)	0	13 (24.0)	41 (76.0)	0	100	-	-
2	53 (98.10)	1 (1.90)	0	48 (88.90)	6 (11.10)	0	90.74	0.462	0.004
3	31 (57.40)	23 (42.60)	0	31 (57.40)	23 (42.60)	0	100	-	-
4	5 (9.30)	41 (75.90)	8 (14.80)	05 (9.30)	41 (75.90)	8 (14.80)	100	-	-
5	38 (70.40)	16 (29.60)	0	39 (72.20)	15 (27.8)	0	94.44	0.864	< 0.001
6	18 (33.30)	36 (66.70)	0	18 (33.30)	36 (66.70)	0	92.60	0.833	< 0.001
7	43 (79.60)	11 (20.40)	0	36 (66.70)	18 (33.30)	0	83.33	0.585	< 0.001
8	0	49 (90.70)	5 (9.30)	2 (3.70)	48 (88.90)	4 (7.40)	94.44	0.702	< 0.001
9	2 (3.70)	43 (79.60)	9 (16.70)	01 (1.90)	44 (81.50)	9 (16.70)	98.15	0.943	< 0.001
10	21 (38.90)	3 (5.60)	30 (55.60)	21 (38.90)	3 (5.60)	30 (55.60)	96.30	0.931	< 0.001
11	45(83.30)	0	9(16.70)	45(83.30)	0	9(16.70)	100	-	-
12	10 (18.50)	44 (81.50)	0	12 (22.20)	42 (77.80)	0	96.23	0.886	< 0.001
13	4 (7.40)	50 (92.60)	0	3 (5.60)	51 (94.40)	0	98.20	0.847	< 0.001
14	4 (7.40)	50 (92.6)	0	3 (5.60)	51 (94.40)	0	98.20	0.847	< 0.001
15	30 (55.60)	24 (44.40)	0	31 (57.40)	23 (42.60)	0	98.16	0.962	< 0.001
16	24 (44.40)	30 (55.60)	0	23 (42.60)	31 (57.40)	0	98.16	0.962	< 0.001
17	47 (87.00)	7 (13.00)	0	47 (87.00)	7 (13.00)	0	100	-	-
18	25 (46.30)	3 (5.60)	26 (48.10)	25 (46.30)	5 (9.30)	24 (44.40)	96.30	0.935	< 0.001
19	27 (50.00)	5 (9.30)	22 (40.70)	27 (50.00)	5 (9.30)	22 (40.70)	100	-	-
20	44 (81.50)	10 (18.50)	0	45 (83.30)	9 (16.70)	0	94.44	0.809	< 0.001
21	53 (98.10)	1 (1.90)	0	52 (96.30)	2 (3.70)	0	98.15	0.658	< 0.001
22	23 (42.60)	31 (57.40)	0	21 (38.90)	32 (59.30)	1 (1.90)	87.40	0.738	< 0.001
23	33 (61.10)	21 (38.90)	0	33 (61.10)	21 (38.90)	0	92.59	0.844	< 0.001
24	44 (81.50)	10 (18.50)	0	45 (83.30)	8 (14.80)	1 (1.90)	90.74	0.685	< 0.001
25	22 (40.70)	4 (7.40)	28 (51.90)	24 (44.40)	4 (7.40)	26 (48.10)	88.89	0.803	< 0.001
26	33 (61.10)	5 (9.30)	16 (29.60)	31 (57.40)	10 (18.50)	13 (24.10)	72.22	0.505	< 0.001
27	18 (33.30)	36 (66.70)	0	17 (31.50)	37 (68.50)	0	87.04	0.704	< 0.001
28	19 (35.20)	35 (64.80)	0	20 (37.00)	34 (63.00)	0	98.15	0.960	< 0.001
29	32 (59.30)	22 (40.7)	0	34 (63.00)	20 (37.00)	0	92.60	0.844	< 0.001
30	23 (42.60)	31 (57.40)	0	24 (44.40)	30 (55.60)	0	94.40	0.887	< 0.001
31	53 (98.10)	1 (1.90)	0	53 (98.10)	1 (1.90)	0	100	-	-
32	0	40 (74.10)	14 (25.90)	0	40 (74.10)	14 (25.90)	96.30	0.904	< 0.001
33	35 (64.80)	19 (35.20)	0	36 (66.70)	17 (31.50)	1 (1.90)	96.30	0.919	< 0.001
34	22 (40.70)	32 (59.30)	0	22 (40.70)	32 (59.30)	0	96.60	0.847	< 0.001
35	3 (5.60)	42 (77.80)	9 (16.70)	2 (3.70)	43 (79.60)	9 (16.70)	98.15	0.947	< 0.001
36	3 (5.60)	43 (79.60)	8 (14.80)	3 (5.60)	43 (79.60)	8 (14.80)	92.30	0.891	< 0.001
37	22 (40.70)	32 (59.30)	0	22 (40.70)	32 (59.30)	0	100	-	-
38	18 (33.30)	7 (13.00)	29 (53.70)	15 (27.80)	9 (16.70)	30 (55.60)	94.44	0.905	< 0.001
39	52 (96.30)	2 (3.70)	0	52 (96.30)	2 (3.70)	0	100	-	-
40	53 (98.10)	1 (1.90)	0	53 (98.10)	1 (1.90)	0	100	-	-
41	18 (33.30)	36 (66.70)	0	18 (33.30)	36 (66.70)	0	96.30	0.917	< 0.001

* *NA - Não se aplica.*

Table 2 illustrates the description of the adherence scores to safe practices for bed bathing identified in the instruments for each evaluator and the interobserver reliability (ICC). The reliability of the instrument was excellent (ICC = 0.962), with a statistically significant correlation (p < 0.001).

**Table 2:** Measures of central tendency and variability for total observer adherence scores and interobserver reliability (ICC). Uberaba, Minas Gerais, Brazil, 2021

Observers	Minimum	Maximum	Average	Median	DP^*^	ICC^α^	*p*
Observer 1	37.14	78.13	56.26	53.22	10.91	0.962	< 0.001
Observer 2	35.00	77.42	55.29	53.24	11.36		

* SD - Standard deviation;

α ICC - Intraclass Correlation Coefficient.

## DISCUSSION

The elaborated instrument is composed of actions that should be performed by the nursing team during the bed bath of the critical patient, aiming at a safe execution, optimization of time, prevention of adverse events, and increase in the quality of care. The instrument went through the processes of apparent and content validation, pretesting, and interobserver reliability to be considered dependable.

Apparent and content validation is the process of accurately examining an elaborate instrument. It is essential to confer validity and reliability, ensuring its operational equivalence, relevance, adequacy, format, context, and mode of application, as well as categorization of responses^(26-27)^.

The study used the Kappa coefficient and the ICC for the checklist validation regarding reliability. It is recommended that Kappa values be higher than 0.60 (substantial agreement) for reliable results^(28)^. According to Santos et al., the ICC is the most suitable psychometric unit to evaluate interobserver correlation^(27)^.

In this study, the Kappa coefficient varied between 0.4 and 0.9, demonstrating moderate to almost perfect agreement, and the ICC was higher than 0.9, evidencing excellent reliability of the instrument. Another complementary analysis conducted was the evaluators’ proportion of agreement, which ranged from 83% to 100%, reinforcing the reliability of the instrument.

A similar study decided to use the percentage of agreement and the calculation of the Kappa coefficient, since the Kappa coefficient separately may present limitations in its results^(29)^.

An investigation conducted to validate a scale that aims to evaluate the nursing care product used interobserver reliability, which assessed 40 evaluations and used Kappa tests to assess agreement and ICC for consistency analysis: ICC ranged from 0.71 to 0.63; and Kappa, from 0.23 to 0.83^(30)^.

Another study aimed to evaluate the interobserver reliability of the Pressure Ulcer Scale for Healing (PUSH) in patients with venous ulcers. It performed forty-six observations and used Kappa and ICC tests. The results showed: a total CHF score >0.9, showing an excellent interobserver reliability; and Kappa values between 0.6 and 0.85, that is, express moderate to a particularly good agreement, respectively^(31)^.

The skin and its microbial flora represent the most significant pathogen reservoir for bloodstream infections^(32)^. The bed bath procedure in the ICU is performed empirically, as there is a lack of standardization of care, and there are few studies on the impacts that bathing causes in critically ill patients^(33)^. In addition, the literature is still scarce regarding the most appropriate technical procedure and the description of actions that would confer a safe procedure to reduce the negative clinical repercussions to patients^(16,18,34-35)^.

In the ICU, most of these events are of the assistance type, making them a major problem. Preventive measures are aimed at decreasing AEs and complications. A study showed that most errors were related to care actions and that continuous training is one of the most effective ways to prevent AEs^(36)^.

Nursing care has been undergoing changes: evidence-based care generates a need for the creation and use of management tools. For this, researchers seek to develop specific instruments, either through cross-cultural adaptations or by improving existing others or creating them, ensuring their validity and reliability. In this sense, the availability of valid and reliable instruments directly interferes with the accuracy of the information collected, influencing the conduct that will be taken^(30,37)^.

The checklists in the health area are created to optimize the collection of information and guide the actions of the procedures, promoting a quick and transparent approach^(38)^. By incorporating protocols, checklists, and instruments based on scientific evidence into nursing care, care is no longer empirical, generating positive results for the team and the patient^(39)^.

A study conducted to assess the use of checklists in the health area evidenced that the ICU is the sector where a higher number of checklists, bundles, and protocols are used due to the critical profile of patients. The main purposes of these instruments are the promotion of quality and safe care, with the checklist the principal tool in the hospital area^(40)^.

### Study limitations

As a limitation of the study is the data collection being conducted during the Covid-19 pandemic, limiting the collection time, and impacting the continuity of the study development for the applicability phase of the instrument in clinical practice for a longer period. However, this limitation did not compromise the response to the objective proposed in this study.

### Contributions to the field

The checklist developed and validated in this study allows that health services evaluate the care provided during bed bathing in critically ill patients, in addition to subsidizing interventions aimed at increasing the safety and quality of care with evidence-based care and standardized and reliable instruments. Thus, with adherence to safe practices and use of standardized tolls in care, patient safety is increased.

## CONCLUSION

The instrument developed in this study (checklist) proved to be dependable when evidencing moderate to almost perfect Kappa values (0.462 to 0.962; p < 0.001) and excellent reliability (ICC = 0.962).

Other investigations are needed with the application of the instrument in a large sample. In addition, the study highlights the possibility of its adoption in critical care units in the setting of Clinical Nursing Practice.
